# Neuroticism and fear of COVID-19 during the COVID-19 pandemic: Testing the mediating role of intolerance of uncertainty and sense of control among Chinese high school students

**DOI:** 10.3389/fpsyg.2022.1010767

**Published:** 2022-12-05

**Authors:** Donghuan Zhang, Min Fan, Lingyi Meng, Xifu Zheng

**Affiliations:** ^1^Key Laboratory of Brain, Cognition and Education Sciences, Ministry of Education, South China Normal University, Guangzhou, China; ^2^School of Psychology, Center for Studies of Psychological Application, and Guangdong Key Laboratory of Mental Health and Cognitive Science, South China Normal University, Guangzhou, China

**Keywords:** teenager, COVID-19, fear, neuroticism, sense of control, intolerance of uncertainty

## Abstract

Since the COVID-19 pandemic broke out in 2019, neuroticism has been proven a predictor of fear of COVID-19 infection. However, only few studies have been conducted on the factors affecting the relationship between neuroticism and this kind of fear. The present study is aimed at analyzing the role intolerance of uncertainty (IU) and sense of control (SOC) play in relation to neuroticism and the fear of COVID-19. We conducted a cross-sectional study in Guangdong and Guangxi provinces, China, and we collected complete datasets from 792 high school students. The main results can be described as follows: (a) individuals with high neuroticism tended to have higher intolerance of uncertainty (IU) and a lower sense of control (SOC); (b) IU and SOC played a mediating role between neuroticism and fear of COVID-19, and a serial mediation effect was found between these factors; (c) after controlling for both IU and SOC, the effect of neuroticism on fear was no longer significant. The results suggested a critical role of IU and sense of control in the causal relationship between neuroticism and fear.

## Introduction

Currently, a pressing matter for countries and regions around the world is coronavirus (COVID-19) prevention and treatment. A period of 2 years have passed since the first COVID-19 case was reported. From then on, China has struggled to keep the virus at bay by implementing the most severe isolation and lockdown. To manage the itinerary of each citizen, the government has prepared an “itinerary card” for each citizen. The itinerary card can automatically obtain information about the places that each citizen has been to through the Internet. When traveling across provinces and cities, citizens need to show their itinerary card to the station. If they have been to an epidemic area within 14 days, regardless of whether they have been infected, they will be restricted. In addition, the government arrested those who refused to report the itinerary information (if they are infected), on the charge of hindering the prevention and treatment of infectious diseases.

China has successfully controlled the epidemic within a certain range for a long time, but it cannot eliminate it. Small-scale epidemics break out from time to time, and these small-scale outbreaks are often related to imported cases. It is difficult to predict when and where the virus will be detected because the incubation period of the virus is relatively long, and the port areas responsible for the entry of foreign personnel are usually crowded. Therefore, with each small-scale outbreak, there are various news reports, increases in travel restrictions, and large-scale case screenings (most of which are mandatory).

One of the psychological factors being discussed worldwide during the COVID-19 pandemic is the fear of COVID-19. With the extremely high infection rate and relatively high mortality rate, individuals apparently began worrying about COVID-19 (Ahorsu et al., 2020), and sometimes, the worry is so great that suicide attempts were reported (Dsouza et al., 2020). Based on the pioneering work by Ahorsu et al. (2020), researchers started to assess the fear of COVID-19 and study its psychological ramification. In previous studies, a positive relationship was found between the fear of COVID-19 and depression, anxiety, and stress (Bakioglu et al., 2020; Rodríguez-Hidalgo et al., 2020; Servidio et al., 2021). Higher fear of COVID-19 was also associated with lower life satisfaction and subjective wellbeing (Bakioglu et al., 2020; Satici et al., 2020), alcohol use (Lee and Crunk, 2020; Montazeri et al., 2020), insomnia (Siddique et al., 2021; Simşir et al., 2021), as well as Internet addiction (Kayis et al., 2021; Servidio et al., 2021) and cyberchondria (Wu et al., 2021). As a relatively stable trait, neuroticism has been proven to predict various mental problems (Barlow et al., 2014). Attempts have been carried out to understand the relationship between neuroticism and fear of COVID-19. For example, researchers found that people with high neuroticism experience more stress, perceive more threats, and feel less efficacy during the pandemic (Liu et al., 2021); neuroticism is positively correlated with the fear of COVID-19 (Caci et al., 2020; Lee and Crunk, 2020). However, we know little about how people with high neuroticism grow to be afraid of COVID-19 (see Caci et al., 2020).

### Neuroticism as a predictor

According to the five-factor model (John and Srivastava, 1999), neuroticism is a stable tendency for people to frequently experience negative emotions. It is associated with many negative psychological consequences (Barlow et al., 2014). Evidence shows that people with high neuroticism tend to experience distress, anxiety, and depression during the pandemic (Abdelrahman, 2020; Lee and Crunk, 2020; Ahmed et al., 2021). This may be because these people experience higher degrees of emotional dysfunction (Yang et al., 2020) and have fewer psychological resources to cope with stress (Zager Kocjan et al., 2021). Moreover, previous studies found that people with high neuroticism also show “threat appeal.” These individuals are more likely to direct attention toward stress-related distracters (Osorio et al., 2003; Verhaak et al., 2004). Similarly, during the pandemic, people with high neuroticism are more likely to develop mental health problems (Modersitzki et al., 2021; Nudelman et al., 2021), talk more about the pandemic in social occasions, think more about the virus (Kroencke et al., 2020), and misinterpret normal bodily sensations as indications of serious disease (Wu et al., 2021). Neuroticism has proved to be a good predictor of the fear of COVID-19 (Caci et al., 2020; Ahmed et al., 2021; Kassim et al., 2022).

### IU as a mediator

As a personality trait, neuroticism may shape individual responses through the cognitive process (McCrae and Costa, 2006). One of the factors that might help explain the neuroticism–fear relationship is intolerance of uncertainty (IU). IU is an individual tendency. People with this tendency cannot tolerate uncertainty and assume that uncertainty usually leads to negative consequences (Carleton et al., 2014). Evidence suggests that high IU is associated with high neuroticism (Berenbaum et al., 2008; Birrell et al., 2011). People with high neuroticism are more likely to avoid ambiguous stimuli than those with low neuroticism (Lommen et al., 2010) and tend to rate uncertain situations more aversive (Hirsh and Inzlicht, 2008). On the contrary, previous studies found that individuals with high IU tend to interpret ambiguous information as threatening and unacceptable (Greco and Roger, 2003; Koerner and Dugas, 2008). These individuals also overestimate the likelihood of negative events (Koerner and Dugas, 2008), even if the perceived probability for negative outcomes is relatively low (Berenbaum et al., 2008).

### Sense of control as a mediator

Another psychological factor that might play a role in neuroticism and the fear of COVID-19 is the sense of control (SOC). From an evolutionary point of view, having a certain sense of control over the environment around oneself is conducive to the survival of the individual (Migone and Liotti, 1998), and the sense of control is very important for life happiness and health (Masters and Wallston, 2005; Park et al., 2008; Bárez et al., 2009). The SOC is negatively predicted by neuroticism (Horner, 1996). Individuals with high neuroticism tend to assume the world is dangerous, and they also believe that they have a hard time coping with the disasters that may happen to them (Barlow et al., 2014). On the contrary, previous studies have found that the lack of control over the environment often plays an important role in the psychological problems associated with stress and anxiety (Bandura et al., 1988). As supported by recent research, a lower sense of control predicted the fear of the pandemic (Imeri et al., 2021) and stress (Khoo et al., 2021).

### The present study

**Figure 1:**
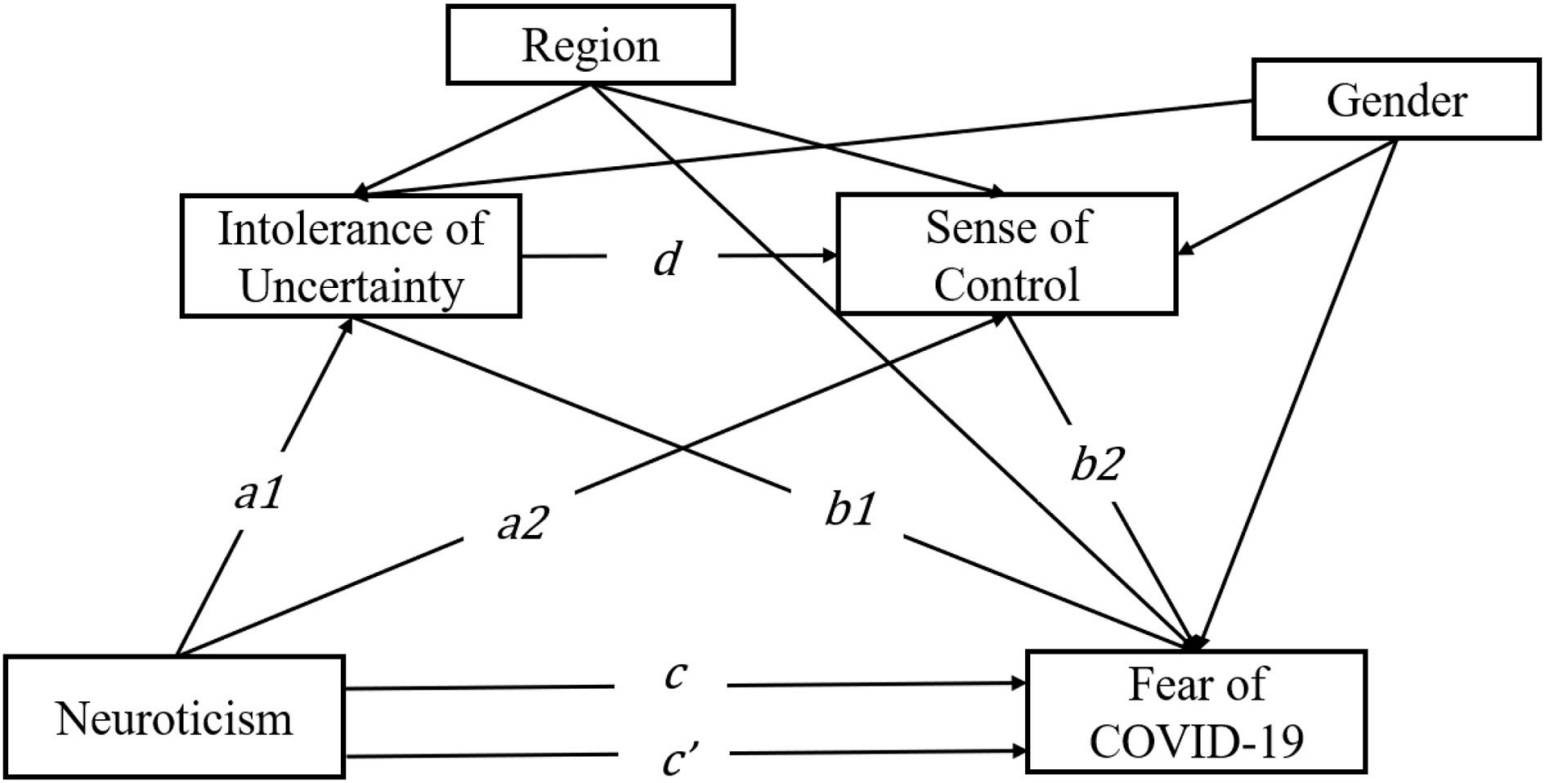
Theoretical model. Intolerance of uncertainty and sense of control as a mediator between neuroticism and the fear of COVID-19. Region and gender were checked as covariates.

Understanding the source of fear may help provide efficient intervention for the vulnerable. Thus, the objective of this study is to construct an IU-SOC model to explain the neuroticism-fear relationship during the COVID-19 pandemic in China. While previous studies have shown that neuroticism is associated with the fear of COVID-19, most of those studies hold an intrinsic standpoint, that is, people with high neuroticism develop fear of the virus because they are inherently fragile and the pandemic environment itself is a stressful situation (Caci et al., 2020; Lee and Crunk, 2020; Liu et al., 2021). However, environmental factors may interact with an individual's trait in a more complex way, rather than simply imposing a “stressful situation” on that person. Therefore, while we build the IU-SOC model based on neuroticism, we further consider cognitive variables that may be associated with the pandemic reality in China. Specifically, we propose an “uncertainty-aversive and control loss” model to explain the relationship between neuroticism and the fear of COVID-19 since the pandemic in China is repetitive and unpredictable. Although strict pandemic prevention strategies have been implemented, the epidemic situation recurred from time to time. Individuals with high neuroticism have lower tolerance toward uncertain situations and a lower sense of control of daily events, which further exaggerate the fear, respectively, because of not knowing and overestimating the harm of the virus. In addition to focusing on the simple mediation effect of these two variables, we are also interested in the potential serial mediating role that these variables play. Since there is evidence that higher IU is associated with a lower sense of control (Buhr and Dugas, 2006; Song and Li, 2019), it is reasonable to speculate that individuals with high neuroticism have lower tolerance toward the uncertain situation, which further leads to a lower sense of control (because of insufficient information acquisition), yielding a higher level of fear toward the COVID-19 pandemic (for theoretical model, see Figure 1). Based on previous studies and the reality of virus spreading, we propose the following hypothesis:

(a) Intolerance of uncertainty mediates the relationship between neuroticism and fear of COVID-19 (paths a1–b1).

(b) Sense of control mediates the relationship between neuroticism and fear of COVID-19 (paths a2–b2).

(c) Intolerance of uncertainty and sense of control play a serial mediating role between neuroticism and fear of COVID-19 (paths a1–d–b2).

## Methods

### Sampling and test procedure

This study adopted a cross-sectional design to collect data from 479 students in Guangdong Province and 313 students in Guangxi Province, China, from 15 October to 15 December 2021, when the COVID-19 situation in the two provinces was relatively stable (age range: 15–17 years, *M* = 15.51; sample size: *N* = 792). A total of four high schools participated in the survey. For each school, we randomly selected four classes from the first grade for testing, that is, 16 of 42 classes from these schools participated in the test. Students were given 20 min to complete the P–P test in the classroom. They were told to keep quiet during the test and follow the instruction:

“In this test, you are going to answer some questions. And these questions do not have a correct answer. Please answer them with your intuition. Only one option can be selected for each question, and please do not select more or omit.”

All students were able to finish the test before timeout. A total of 56 students were excluded from data analysis due to incorrect answering to screening items (three items, e.g., “Please choose “agree” for this question,” and choosing otherwise was seen as an incorrect response). Another 31 students were excluded from the analysis because no gender information was provided. This reduced the number of participants to 705, with female students (*n* = 314) counting for 44.5% of the total participants. Multiple linear regression was calculated using G^*^Power to validate the sample size (α = 0.025, power = 0.8, Cohen *f*^2^ = 0.02), with three predictors. The desired total sample size was 651. The study was approved by the Ethics Committee of South China Normal University. The study was conducted under the supervision of the school officials and was given consent by school officials as a part of the general psychological survey. Informed consent was provided by participants and their guardians.

### Measures

#### Demographics

Participants' sociodemographic characteristics, including gender, age, and region, were collected. Due to legal concerns and demands proposed by the participating schools, names and socioeconomic status were not collected.

#### Fear of COVID-19 scale (FCS-19 Chinese version)

Students' fear of the COVID-19 pandemic was accessed by the fear of COVID-19 scale (FCS-19). The FCS-19 was developed by Ahorsu et al. (2020), and the Chinese version of the scale was revised by a Chinese scholar (Chi et al., 2022). The FCS-19 includes seven items and a two-factor structure that assess the cognition–emotion aspect of the fear of COVID-19 by items such as “It makes me uncomfortable to think about coronavirus-19,” and the physical aspect of the fear of COVID-19 by items such as “My hands become clammy when I think about coronavirus-19.” The items were scored on a five-point Likert scale (1 = “strongly disagree,” 3 = “neutral,” and 5 = “strongly agree”). The total score was calculated by summing up the score of every item, and Cronbach's α value of the scale was 0.92 (Chi et al., 2022).

#### Chinese big five personality inventory brief version (CBF-PI-B)

Neuroticism was measured by using the neuroticism subscale of the short form CBF-PI (Chinese version), which was adapted from the NEO-PI-R. The CBF-PI Chinese version has 40 items in total, and the neuroticism subscale has eight items. The Cronbach's α value of the neuroticism subscale was 0.814 (Wang et al., 2011). The items were scored on a five-point Likert scale. Item 4 was scored reversely, and the total score was calculated by summing up the score of every item.

#### Intolerance of uncertainty scale (IUS-12 Chinese version)

Intolerance of uncertainty was measured by the intolerance of uncertainty scale (IUS-12, Chinese version), which was developed by Carleton et al. (2007) and adapted to the Chinese population by (Wu et al., 2016). The IUS-12 Chinese version includes 12 items, and the scoring method also follows a five-point Likert scale. The reliability of IUS-12 Chinese version is acceptable (Cronbach's α = 0.83) (Wu et al., 2016). The total score was calculated by summing up the score of every item. Higher scores indicate greater intolerance of uncertainty (Wu et al., 2016).

#### Sense of control scale (SOCS)

The sense of control scale was designed to measure the extent to which an individual feels competent and constrain-free about happenings in their daily lives (Lachman and Weaver, 1998). The scale comprises two dimensions: personal mastery and perceived constraints (Lachman and Weaver, 1998). Personal mastery (four items) indicates how people perceive their efficacy in doing something, and perceived constraints (eight items) indicate the extent to which one believes there are things beyond one's control. Cronbach's α values of the two subscales were 0.7 and 0.86, respectively (Lachman and Weaver, 1998). This scale was translated into Chinese by Li (2012), and Cronbach's α value of the adapted Chinese version was 0.79. The items were scored on a five-point Likert scale. To obtain the total score of the SOCS, all items of the “perceived constraints” subscale were scored reversely, and then, the two subscales were standardized and summed (Kraus et al., 2009).

### Data analysis

Data were analyzed by SPSS software (IBM, SPSS Statistics, version 21). Two-way ANOVAs and correlation analysis were performed before modeling. For some variables, the main effects of gender and region are significant (Table 1). Thus, these two variables were checked as covariates in the model. In addition, correlation analysis showed that some of the variables did not follow a normal distribution (Table 2). Thus, a bootstrap method was adopted, based on previous studies (Shrout and Bolger, 2002). A total of 5,000 bootstrap samples were created for analysis.

**Table 1:** Description statistics and ANOVA.

**Region**	**Gender**	***n***	***N***	**IU**	**SOC**	**MA**	**PC**	**FOC**
1	Male	178	22.81 ± 6.65	36.59 ± 7.48	34.36 ± 5.23	13.68 ± 2.82	20.68 ± 5.38	15.06 ± 6.17
	Female	136	24.78 ± 6.48	36.27 ± 6.64	35.29 ± 4.88	13.72 ± 2.11	21.57 ± 5.34	14.33 ± 4.65
2	Male	256	26.08 ± 6.95	37.80 ± 6.93	35.62 ± 5.42	13.15 ± 2.55	22.47 ± 5.65	16.74 ± 5.71
	Female	135	26.53 ± 6.18	37.46 ± 6.63	35.58 ± 4.44	13.55 ± 2.40	22.03 ± 5.26	16.94 ± 4.70
*F* (region)			5.524	0.365	1.288	1.253	0.286	0.379
*p* (region)			**0.019**	0.546	0.257	0.263	0.593	0.538
*F* (gender)			23.79	4.75	3.87	3.12	7.13	24.8
*p* (gender)			**<0.001**	**0.030**	**0.049**	0.078	**0.008**	**<0.001**
*F* (interaction)			2.18	<0.001	1.51	0.85	2.51	1.17
*p* (interaction)			0.141	0.984	0.220	0.356	0.114	0.281

N, neuroticism; IU, intolerance of uncertainty; SOC, sense of control; MA, perceived mastery; PC, perceived constraints; FOC, fear of COVID-19; *p* < 0.05 is shown in bold.

**Table 2:** Descriptive statistics and correlations among study variables.

**Variable**	**1**	**2**	**3**	**4**	**5**
1. Fear of COVID-19	–				
2. Neuroticism	0.272^**^	–			
3. Intolerance of uncertainty	0.284^**^	0.586^**^	–		
4. Personal mastery	−0.078^*^	−0.250^**^	−0.080	–	
5. Perceived constraints	0.308^**^	0.623^**^	0.531^**^	−0.365^**^	–
α	0.87	0.88	0.81	0.70	0.82
*M*	15.77	24.76	37.04	13.54	21.63
*SD*	5.65	6.78	7.08	2.551	5.464
Skewness	0.433	−0.043	0.010	−0.292	0.044
Kurtosis	−0.068	−0.420	0.502	1.128	−0.225

** *p* < 0.01,

* *p* < 0.05.

The PROCESS macro for the SPSS (Model 6) (Hayes, 2013) was used to analyze the model, with intolerance of uncertainty and sense of control as mediators. However, when the factor “sense of control” was included in the serial mediation model, the serial mediation effect for paths a1–d–b2 was not significant. Therefore, we used “perceived constraints (PC),” one of the sub-dimensions, as a substitute for the sense of control. Cronbach's α value of the two subscales in this study are presented in Table 2, together with other scales. The internal consistency of the “perceived constraints” is acceptable (α = 0.82). We will present the main result of the original model in the Mediation Analyses section and discuss the reason for model modification in the Discussion section. For charts and more details, see Supplementary Appendix.

## Result

### Preliminary analyses

Two-way ANOVAs were performed to detect differences between region and gender on variables of interest. As shown in Table 1, the main effect of the region was significant for neuroticism [*F*_(1, 701)_ = 5.52, *p* = 0.019], and the main effects of gender were significant for all variables of interest [lowest *F*_(1, 701)_ = 4.75], except “perceived mastery” [*F*_(1, 701)_ = 3.12, *p* = 0.078). No significant interaction effects were observed on any variables of interest.

The correlation coefficients and scale-wise descriptive statistics are presented in Table 2. All the variables show significant inter-correlation with each other. Multicollinearity bias and common method bias were also checked. The variance inflation factor values were between 1.10 and 2.07, and the tolerance values were between 0.48 and 0.94, suggesting that there is no multicollinearity problem for the data.

### Mediation analyses

First, the full sense of control scale was introduced into the model. As expected, neuroticism is a good predictor of the fear of COVID-19 (total effect, *B* = 0.254, 95% CI = 0.181–0.324, *p* < 0.001), and a parallel mediation effect was found with IU (*B* = 0.093, 95% CI = 0.036–0.155, *p* = 0.002) and SOC (*B* = 0.049, 95% CI = 0.005–0.094, *p* = 0.035). However, we found no sequential mediation effect between neuroticism and the fear of COVID-19 (*B* = 0.004, 95% CI = −0.002–0.012, *p* = 0.281). Moreover, when the two mediators were introduced into the model, the direct effect was still significant (*B* = 0.107, 95% CI = 0.011–0.202, *p* = 0.029) (for details, see Supplementary Appendix). As a result, “perceived constraints,” one of the sub-dimensions, was used as a substitute for the sense of control.

**Figure 2:**
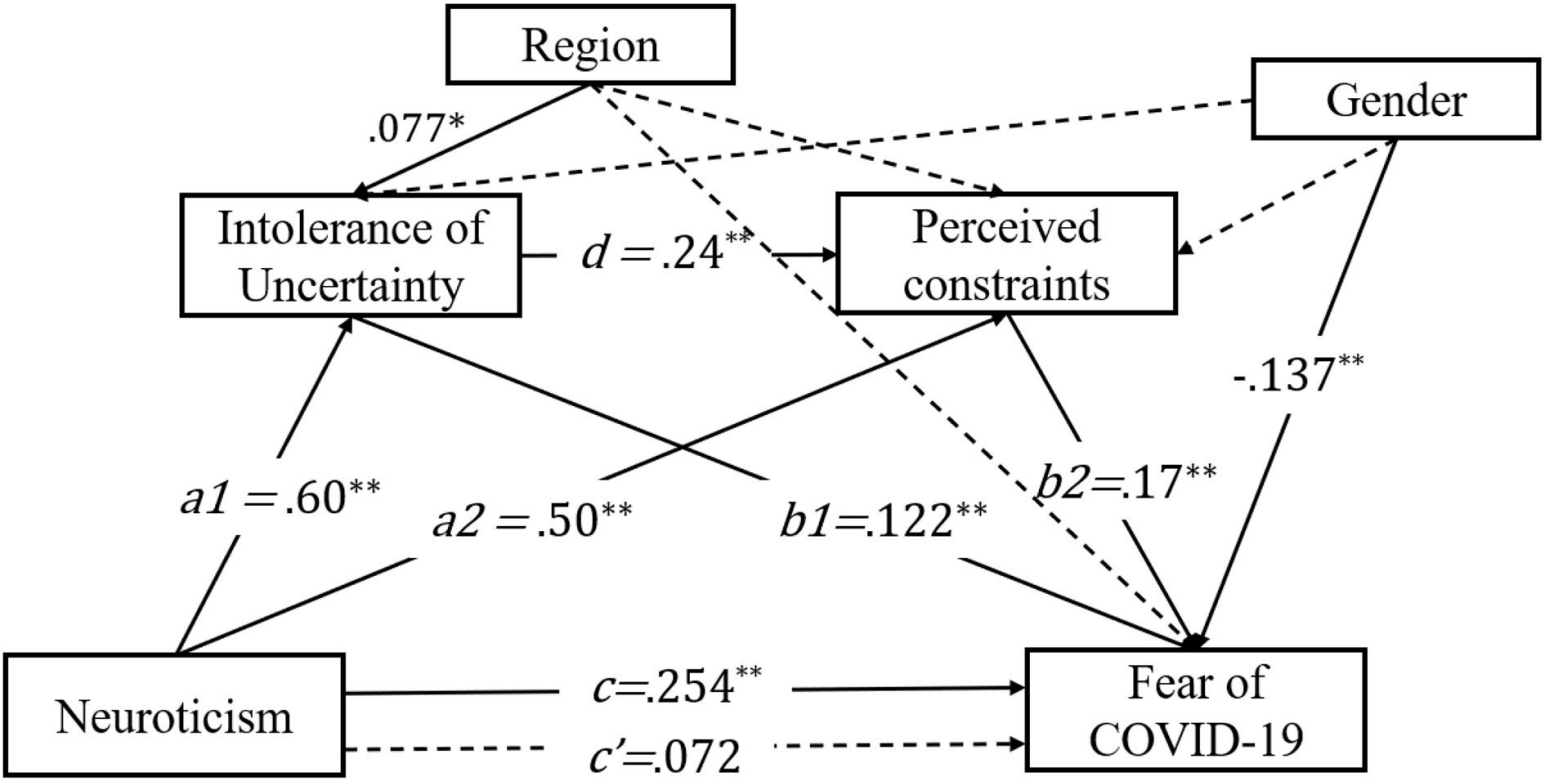
Result of serial multiple mediational model. **p < 0.01, *p < 0.05. Values shown are standardized coefficients. Region and gender were checked as covariates.

The results given in the following text are the results of the modified model. The paths analyzed are presented in Figure 2. Consistent with the previous research (Abdelrahman, 2020; Caci et al., 2020; Ahmed et al., 2021; Liu et al., 2021), neuroticism was a good predictor of the fear of COVID-19 (total effect, *B* = 0.254, 95% CI = 0.181–0.324, *p* < 0.001). However, when mediators were introduced into the model, this coefficient decreased greatly, and it was no longer significant (direct effect, *B* = 0.072, 95% CI = −0.027–0.170, *p* = 0.157).

Table 3 shows the result of the mediation effect analysis. Neuroticism was an indirect predictor of the fear of COVID-19 through IU (*B* = 0.073, 95% CI = 0.014–0.134, *p* = 0.017). Likewise, neuroticism also indirectly predicted the fear of COVID-19 through PC (*B* = 0.085, 95% CI = 0.035–0.139, *p* = 0.001). Neuroticism indirectly predicted the fear of COVID-19, through IU and perceived constraints, in a sequential manner (*B* = 0.024, 95% CI = 0.009–0.044, *p* = 0.006). In general, the indirect effect counted for 72% of the total effect. The simple mediation effect of IU, PC, and their serial mediation effect accounted for 28.7, 33.4, and 9.4% of the total effects, respectively. The direct effect was not supported by this result (*B* = 0.072, 95% CI = −0.027–0.170, *p* = 0.157).

**Table 3:** Indirect effect of neuroticism on fear of COVID-19 *via* intolerance of uncertainty and perceived constraints.

**Path**	**Effect**	***p*-value**	**95%CI**	
			**LL**	**UL**
N → IU → FOC	0.073	0.017	0.015	0.134
N → PC → FOC	0.084	0.001	0.034	0.138
N → IU → PC → FOC	0.024	0.006	0.009	0.044
Total effect	0.253	<0.001	0.181	0.324
Direct effect	0.071	0.157	−0.027	0.170
Total indirect effect	0.183	<0.001	0.105	0.262

CI, confidential interval; LL, lower limit; UL, upper limit; N, neuroticism; IU, intolerance of uncertainty; PC, perceived constraints; FOC, fear of COVID-19.

## Discussion

Although neuroticism is a good predictor of the fear of COVID-19, few models link these two factors with individual cognitive differences, let alone the actual pandemic situation. Through mediation analysis, the present study tried to explain this link with intermediate variables such as intolerance of uncertainty and sense of control (or perceive constraints). As a cognitive style, these variables may reflect the interaction of personality and social environment and bridge the gap between the psychological structure and event-related emotions. Under the assumption of the model, students with high neuroticism were more likely to display the fear of COVID-19. This may be related to two cognitive factors: a higher level of intolerance of uncertainty and a lower level of sense of control.

First, neuroticism significantly predicted the fear of COVID-19, as is consistent with previous research during the pandemic (Caci et al., 2020; Ahmed et al., 2021; Kassim et al., 2022). This is also consistent with previous studies suggesting that people with high neuroticism show more fear of negative information such as loss (Blackwell et al., 2017), pain (Goubert et al., 2004), and death (Loo, 1984; Pérez-Mengual et al., 2021). For these people, fear may be caused by higher emotional reactivity and less stress coping resources (Larsen and Ketelaar, 1991). Therefore, it is reasonable for them to experience fear to a greater extent during the pandemic situation (Gunthert et al., 1999). However, the epidemic environment is complex and not just a simple “stress situation,” and the intrinsic theory is not sufficient to explain the relationship between neuroticism and the fear of the COVID-19 pandemic. Cognitive factors that are related to both personality and reality need more attention.

As expected, IU played a mediating role between neuroticism and the fear of COVID-19. This is consistent with previous studies suggesting that IU mediates the relationship between neuroticism and worry (Yang et al., 2015). As a cognitive variable, IU was conceptualized as originating as the result of personality, particularly neuroticism (Fergus and Rowatt, 2014), and it was regarded as the “core lower order factor” of neuroticism (Norton and Asmundson, 2007). People with high neuroticism are sensitive to uncertainty. They use lower criteria for detecting danger in the face of ambiguous stimuli (Lommen et al., 2010) and find it difficult to endure uncertain situations (Berenbaum et al., 2008; Birrell et al., 2011). There are many uncertainties about the current epidemic situation in China: (1) Although the domestic epidemic situation is well controlled, there are related infections caused by imported cases; (2) there are illegal immigrants in the border area or residents in the border area contact with overseas cases; (3) some people who are asymptomatic might hide their itinerary to avoid official inspection, and (4) the virus itself is difficult to eliminate. As a result, individuals with high neuroticism may often find themselves in a “dangerous and uncertain” environment in which there are many opportunities to be exposed to the virus.

Our next hypothesis has also been confirmed. The sense of control (and its sub-dimension “perceived constraints”) played a mediating role in neuroticism and the fear of COVID-19. People with higher neuroticism tended to report a lower sense of control (Horner, 1996). A period of 2 years have passed since the first COVID case was reported. The process of epidemic prevention and control is tortuous with virus mutation and relapse. People with higher neuroticism can reckon that virus is unstoppable and uncontrollable, leading to overestimation of the harm and greater fear of the COVID-19 pandemic. Another explanation may be related to the mandatory isolation policy. According to Modersitzki et al. (2021), individuals with neuroticism perceived work measures and political restrictions as more limiting. In this case, the fear of COVID-19 is not only the fear of infection itself but also the fear of its following social ramification and legal reliability. During isolation, people lose their liberties and necessary social contact. “To be a patient, to be in prison.” The idea of totally handing out one's control of life may be overwhelming for people with a lower sense of control, especially when they believe there is extraordinary power outside them and from which the offense cannot be undone.

The third hypothesis of our study has been partly confirmed. First, we introduced the full sense of control scale into the model and found that the serial mediation effect for IU and SOC was insignificant. IU was not a good predictor of the SOC. This may be because the personal mastery dimension of the SOCS was less relevant to the topic of this study (e.g., to believe the virus is predictable and preventable). Items in this subscale such as “Whether or not I can get what I want is in my own hands” and “When I really want to do something, I usually find a way to succeed at it” focus more on people's attitude toward achievement. This is supported by correlation analysis shown in Table 2. The factor “perceived constraints” correlated with the fear of COVID-19 (*r* = 0.30, *p* < 0.001) and IU (with *r* = 0.53, *p* < 0.001) in a more explicit manner, while “personal mastery” correlated with fear of COVID-19 (with *r* = −0.078 and *p* = 0.035), but not IU (with *r* = −0.08, *p* = 0.31). People with high IU may search for information to reduce their uncertainty during the pandemic, but uncertainty-reducing behaviors may lead to higher perceived threat severity (Wu et al., 2021). However, when the pandemic is difficult to predict, people often cannot ensure they are insulated from infection (i.e., they may not be able to end their uncertainty). As a result, the threat appraised increases. For those with high perceived constraints, the inability to obtain the information they want or prepare for the coming danger may make them more vulnerable to fear.

## Limitations

The main limitation to this study is its cross-sectional design, where cause-and-effect solution must be derived with caution. For example, it may difficult to decide which variable goes first when it comes to IU and SOC (or PC). It may be that individuals with high IU rate the pandemic environment as more threatening, which further compromises their control of life, or individuals with a lower SOC are more sensitive to uncertainty. Moreover, other factors such as family environment, parenting style, and social media use may explain the correlations between variables of interest. Social factors may shape one's attitude toward viruses, uncertainty, self-efficacy, and even personality.

The second limitation is that the population included in this study (all of them are high school students and come from two different regions) may restrict the generalization of the results. It is argued that many high schoolers inherently have less control of their lives relative to adults, and their personality traits or trait-like variables are unstable. Future research should pay more attention to the differences between adults and adolescents on these variables. In addition, since the samples of this study are from two different provinces in China, there could be potential difference in the explanatory power of the model used in these regions. Thus, future studies should use a mixed linear model to detect such differences.

Finally, it has been over 2 years since the first infection was reported. People may grow apathetic in this protracted wrestle with the virus, so it is reasonable to expect public fear of COVID-19 to decrease, but it is still unclear how this will influence the present study results.

## Conclusion

In general, our study indicated a mediating role for IU and PC in neuroticism and the fear of COVID-19. IU and PC are mainly responsible for the fear of COVID-19 among high school students with neuroticism. Although this study has certain limitations, we believe that the results of the current study have deepened our understanding of the individual differences of the fear of COVID-19 and will help mental health practitioners develop treatment programs for these people during the pandemic situation. Treatment programs in mental health should aim to help these people establish a correct understanding of the virus and regain their control.

In addition, the results of this study may also bring some warnings to public governance. News reports and official propaganda, which emphasize the transmission ability of the virus and the serious consequences of infection, and which aim to remind people to pay attention to daily epidemic prevention, may cause fear (Tannenbaum et al., 2015; Pakpour and Griffiths, 2020), especially for the vulnerable. The government should provide the public with objective information about the virus and give scientific medical advice, if needed. The use of threats by exaggerating the harm of the virus may cause real harm.

## Data availability statement

The raw data supporting the conclusions of this article will be made available by the authors, without undue reservation.

## Ethics statement

The studies involving human participants were reviewed and approved by Human Research Ethics Committee for Non-Clinical Faculties, The School of Psychology, South China Normal University. Written informed consent to participate in this study was provided by the participants' legal guardian/next of kin.

## Author contributions

XZ: conceptualization and methodology. DZ: investigation, data curation, and writing—original draft preparation. MF: supervision and writing—reviewing and editing. LM: writing—reviewing. All authors contributed to the article and approved the submitted version.

## Funding

This work was supported by the National Natural Science Foundation of China (31970996), Major Program of the National Social Science Foundation of China (19ZDA360), and psychological services and counseling bases for the Happy Guangzhou project, which received funding from the Guangzhou government.

## Conflict of interest

The authors declare that the research was conducted in the absence of any commercial or financial relationships that could be construed as a potential conflict of interest.

## Publisher's note

All claims expressed in this article are solely those of the authors and do not necessarily represent those of their affiliated organizations, or those of the publisher, the editors and the reviewers. Any product that may be evaluated in this article, or claim that may be made by its manufacturer, is not guaranteed or endorsed by the publisher.
