# Supplementary material for: Neuroticism and fear of COVID-19 during the COVID-19 pandemic: Testing the mediating role of intolerance of uncertainty and sense of control among Chinese high school students
Source: Front Psychol. 2022 Dec 5;13:1010767. doi: 10.3389/fpsyg.2022.1010767 (PMC9760983; doi:10.3389/fpsyg.2022.1010767)
Supplement: Supplementary file 1 [file Data_Sheet_1.PDF]

Appendix

At first, we introduced the full sense of control scale into the model. The correlation coefficients and descriptive statistics of variables are presented in **Table 1**. All of the variables showed significant intercorrelation with each other.

Table 1

Descriptive statistics and correlations among study variables.

Variable	1	2	3	4
1. fear of COVID-19	-			
2. neuroticism	0.27**	-		
3. intolerance of uncertainty	0.29**	0.59**	-	
4. sense of control	-0.23**	-0.50**	-0.34**	-
Cronbach's α	0.87	0.88	0.81	0.82
M	15.77	24.76	37.04	0
SD	5.65	6.78	7.08	1.70
Skewness	.433	-.043	.010	-.132
Kurtosis	-.068	-.420	.502	.229

** $p < .001$.

As is expected, neuroticism is a good predictor of fear of COVID-19 (Total effect, $B = 0.254$, 95%CI = 0.181~0.324, $p < .001$). IU ($B = 0.093$, 95%CI = 0.036~0.155, $p = .002$) and SOC ($B = 0.049$, 95%CI = 0.005~0.094, $p = .035$) mediate between neuroticism and COVID-19 fear respectively. However, the serial mediating effect for IU and SOC is not significant ($B = 0.004$, 95%CI = -0.002~0.012, $p = .281$) (see **fig. 1** and **table 2**). Moreover, after introducing the two mediator into the model, the direct effect was still significant ($B = 0.107$, 95%CI = 0.011~0.202, $p = .029$).

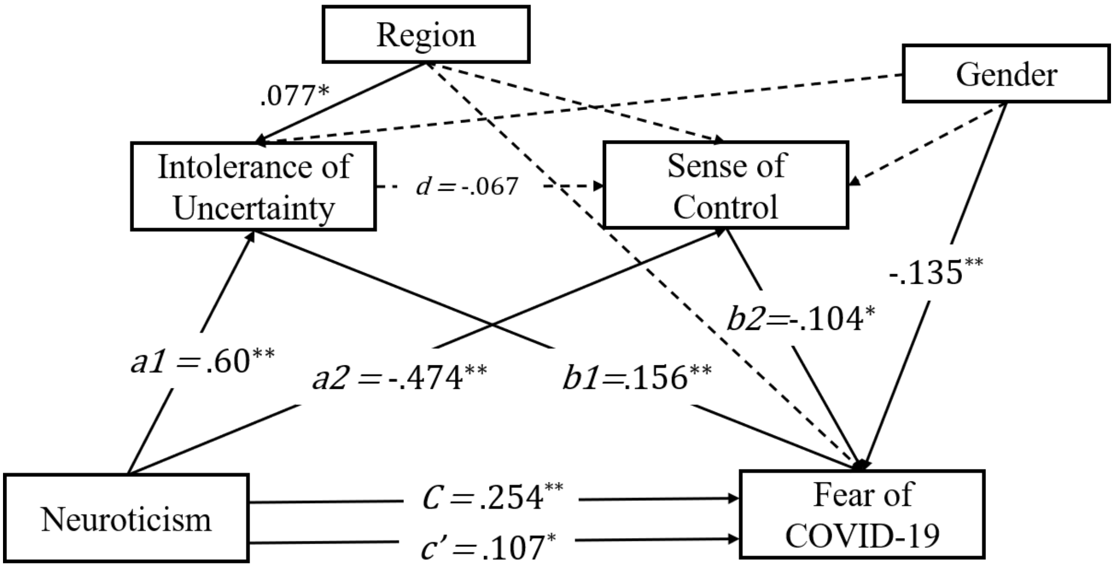

Fig. 1. The serial multiple mediational model result, ** $p < .001$, * $p<.05$ Values shown are standardized coefficients.

Table 2

The indirect effect of neuroticism on fear of COVID-19 via intolerance of uncertainty and perceived constraint.

Path	Effect	*p-value*	95%CI	
			LL	UL
N→IU→FoC	0.094	.002	0.035	0.156
N→SOC→FoC	0.049	.035	0.006	0.094
N→IU→SOC→FoC	0.004	.281	-0.002	0.013
Total effect	0.254	<.001	0.181	0.324
Direct effect	0.106	.029	0.011	0.202
Total indirect effect	0.146	<.001	0.072	0.224

Note. CI confidential interval; LL lower limit; UL upper limit; N neuroticism; IU intolerance of uncertainty; PC perceived constraint; FoC fear of COVID-19.
